# Draft Genome Sequence of *Streptomyces olivochromogenes* NBRC 3561, a Bioactive Peptide-Producing Actinobacterium

**DOI:** 10.1128/genomeA.01048-17

**Published:** 2017-09-28

**Authors:** Hideo Dohra, Yuto Miyake, Shinya Kodani

**Affiliations:** a Instrumental Research Support Office, Research Institute of Green Science and Technology, Shizuoka University, Suruga-ku, Shizuoka, Japan; b Department of Biological Science, Graduate School of Integrated Science and Technology, Shizuoka University, Suruga-ku, Shizuoka, Japan; c Department of Applied Biological Chemistry, Graduate School of Integrated Science and Technology, Shizuoka University, Suruga-ku, Shizuoka, Japan

## Abstract

Recently, we found that *Streptomyces olivochromogenes* NBRC 3561 produced a bioactive peptide, so we sequenced its genome to clarify its biosynthesis. We report here the draft genome sequence of *S. olivochromogenes* NBRC 3561, in which 40 potential secondary metabolite gene clusters were predicted by antiSMASH.

## GENOME ANNOUNCEMENT

*Streptomyces* is the largest genus in the phylum *Actinobacteria*, and its members are prolific producers of bioactive secondary metabolites, as has been well studied (1). *Streptomyces olivochromogenes* produces useful enzymes, such as xylose isomerase. However, there have been few reports on low-molecular-weight compounds of *S. olivochromogenes*. Recently, our chemical investigation revealed that *S. olivochromogenes* NBRC 3561 produced a bioactive peptide. The draft genome sequence of *S. olivochromogenes* NBRC 3561 was determined and searched for a gene cluster possibly involved in the production of the bioactive peptide.

*S. olivochromogenes* strain NBRC 3561 was acquired from the NITE Biological Resource Center, Japan. Genomic DNA of *S. olivochromogenes* NBRC 3561 was extracted using the DNeasy blood and tissue kit and fragmented using the Covaris Acoustic Solubilizer. A paired-end library constructed by the TruSeq DNA PCR-free library preparation kit was sequenced using the Illumina MiSeq platform (301-bp paired ends). The raw read sequences were cleaned up using Trimmomatic (2) by trimming adapter sequences, low-quality ends (quality score, <15), the last 301 bases, and reads less than 150 bp. The filtered reads were additionally processed using khmer version 2.0 (3) by filtering reads with a low *k*-mer coverage (<3) to remove sequencing errors and contaminated sequences. The resultant 3,567,049 high-quality reads totaling 764 Mb, which corresponds to an approximately 66-fold coverage of the genome, were assembled using SPAdes version 3.10.0 (4) with a *k*-mer size of 21, 33, 55, 77, 99, and 127 bp and the options --careful, --only-assembler, and --cov-cutoff auto, and contigs less than 200 bp were eliminated.

The draft genome of *S. olivochromogenes* NBRC 3561 contains 131 contigs consisting of 11,639,641 bp with a G+C content of 70.22%. The draft genome was annotated using the DFAST Legacy server (5) based on Prokka (6) with a RefSeq database. The *S. olivochromogenes* NBRC 3561 genome contains 10,550 protein-coding sequences and 88 tRNAs. The proteins encoded by the genome were additionally annotated with the COG (Clusters of Orthologous Groups) database (7). Among 10,550 proteins, 6,621 (62.8%) were assigned to COG functional categories, including 82 proteins involved in the ABC-type multidrug transport system (COG0842, COG1131, and COG1132) and 31 proteins involved in the ABC-type antimicrobial peptide transport system (COG0577 and COG1136). This result suggests that *S. olivochromogenes* NBRC 3561 has developed transport systems for bioactive peptides such as antimicrobial peptides.

Prediction of secondary metabolite biosynthesis gene clusters by antiSMASH version 3.0.5 (8) suggested that the *S. olivochromogenes* NBRC 3561 genome contained 40 potential biosynthetic gene clusters, including 7 polyketide synthases (PKSs), 4 nonribosomal peptide synthetases (NRPSs), 4 bacteriocins, 4 terpenes, 3 siderophores, 2 lantipeptides, and a lassopeptide. Among 40 gene clusters, 5 were predicted to be hybrid clusters, such as an NRPS-PKS hybrid and a lantipeptide-terpene hybrid.

The genome information and predicted secondary metabolite biosynthesis gene clusters of *S. olivochromogenes* NBRC 3561 will contribute to studies on the structure and function of bioactive compounds and their biosynthetic pathways and transport systems.

### Accession number(s).

The draft genome sequence of *S. olivochromogenes* NBRC 3561 was deposited in DDBJ/EMBL/GenBank under accession no. BDQI00000000. The version described in this paper is the first version, BDQI01000000.
